# Burden and risk factors of invasive group B Streptococcus disease among neonates in a Chinese maternity hospital

**DOI:** 10.1186/s12879-018-3660-1

**Published:** 2019-02-06

**Authors:** Qunhua Ying, Shutan Wang, Xiuming Lou, Jinlong Ding, Jiefeng Ding

**Affiliations:** 1 Department of Clinical Laboratory, Shaoxing Women and Children’s Hospital, 305 East Street Road, Shaoxing, 312000 Zhejiang China; 2 University of International Business and Economics, 10 Huixing East Road, Beijing, 100029 China

**Keywords:** Streptococcus group B (GBS), Neonatal Sepsis, GBS risk factors, Early-onset disease (EOD), Late-onset disease (LOD)

## Abstract

**Background:**

There is a lack of data regarding the prevalence of invasive group B streptococcus (GBS) infection among neonates in China. This study aimed to investigate the incidence and mortality of invasive GBS infection and to identify the risk factors in our hospital.

**Methods:**

Seventy-four cases admitted between January 2011 and December 2016 was included in this study. A retrospective matched case-control study was conducted in a tertiary maternity and paediatric hospital. Risk factors for the acquisition of invasive GBS infection and mortality were analysed by univariable and multivariable analysis.

**Results:**

We collected and analysed data from 74 infants aged < 3 months with invasive GBS infection. Among 67,985 live births, we calculated an incidence of 1.09 per 1000 live births (95%CI:0.81–1.37%); the incidence of Early-onset GBS disease (EOD, *n* = 65) and Late-onset GBS disease (LOD, *n* = 9) were 0.96‰(95%CI:0.73–1.19%) and 0.13‰(95%CI:0.04–0.22%), respectively. Overall, pneumonia accounted 63.1% (41/65) of EOD, and sepsis accounted 88.9% (8/9) cases of LOD, respectively. The overall case fatality rate was 8.11% (6/74), including 7.69% (5/65) among cases of EOD and 11.1% (1/9) among cases of LOD. No predictor of mortality was found. Membrane stripping (*P* = 0.005, aOR: 3.68, 95% CI: 1.48–9.13) and non-resident mother (*P* < 0.001, aOR: 5.88, 95% CI: 2.36–14.61) were independent risk factors for EOD; no increased risk was found for LOD.

**Conclusions:**

This study demonstrates remarkable country-specific variation in comparison with other countries. Our findings can improve awareness of neonatal GBS infection and lay a cornerstone to ensure accurate representation of the burden.

## Background

Group B Streptococcus (GBS) is a leading cause of invasive neonatal infection [1]. There are several previous studies of the burden and risk factors for invasive GBS disease in many countries [2–4]. However, there is a lack of data on the prevalence of invasive GBS infection among neonates in China. China’s new two-child policy will result in more infants being born each year; therefore, reducing neonatal GBS infection is important. Hence, we performed a retrospective study to evaluate the risk factors and burden for invasive neonatal GBS infection in our hospital in east China.

## Methods

### Study setting

We undertook a retrospective case-control study to evaluate the incidence, mortality, risk factors, and characteristics associated with invasive neonatal GBS infection. Cases were treated at Shaoxing Maternity and Child Care Hospital, a 600-bed modern hospital specializing in Gynaecology, Obstetrics and Paediatrics in Shaoxing, Zhejiang Province in east China. Approximately 10,000 infants are delivered each year, and GBS screening and intrapartum antibiotic prophylaxis (IAP) during pregnancy is not standard of care. Risk factors were assessed by comparing infants with invasive neonatal GBS infection to those without, and variables assessed as possible risk factors for invasive GBS disease included previously identified risk factors, such as preterm delivery (< 37 weeks’ gestational age), prolonged rupture of membranes (> = 18 h), birth weight < 2500 g, known genital GBS colonization, intrapartum fever, and non-use of IAP. Resident status (mothers whether or not being resident of Shaoxing) and membrane stripping (women who did and did not undergo membrane stripping) were also assessed.

### Study design and population

Cases of GBS infection were defined as infants < 90 days of age in whom GBS was isolated from blood or cerebrospinal fluid (CSF) and otherwise sterile body fluids. Investigation was performed from January 1, 2011 through December 31, 2016.Early onset disease (EOD) was defined when GBS was isolated in infants younger than 7 days of life, and infants 7–89 days of age with GBS disease were regarded as having late-onset disease (LOD) [5]. Cases were identified by medical records investigation of the paediatric wards and microbiology services. For each neonate with invasive GBS infection, we randomly selected two controls for cases of EOD and 3 controls for cases of LOD from infants who were born within the same period and had no bacterial infection.

### Microbiological identification and susceptibility testing

Blood and throat swab cultures are routinely performed in infants admitted to the neonatal intensive care unit (NICU) with suspected sepsis or pneumonia. Cerebrospinal fluid (CSF) and other specimen cultures are performed in infants with suspected meningitis or other clinical manifestations. GBS was isolated from blood samples using the BACT/ALERT 3D microbial detection system (bio-Mérieux, Marcy-l’Étoile, France). Positive blood culture bottles were subsequently sub cultured to Columbia blood agar (bio-Mérieux, Marcy-l’Étoile, France) incubated aerobically at 35 °C under 5–10% CO_2_ and observed for colony growth for 48 h. CSF samples were inoculated into 3D blood culture bottles and immediate Gram stain was performed. Other swabs such as eye swab and umbilical exudation swab were plated onto Columbia blood agar plates that were incubated at 35 °C for 18–24 h under 5–10% CO_2_ and examined for growth of ß-haemolytic GBS-like colony morphology. Presumptive identification of GBS was based on Gram stain and a positive CAMP test, determined by an arrowhead-shaped zone of complete haemolysis. Vitek 2 COMPACT (bio-Mérieux, Marcy-l’Étoile, France) was used to perform identification and susceptibility testing.

### Data collection

Data were obtained from medical records, and relevant data were recorded on questionnaires. During the study period the one-child policy was in place and as such may effect risk calculation.Only 2 mothers of cases had previous children, each of whom died due to pneumonia of unknown pathogen.

### Statistical analysis

Continuous variables were compared with the Student t-test (for normally distributed variables) or the Mann–Whitney U test (for non-normally distributed variables) and are presented as the mean ± standard deviation (SD) or median (range). Categorical variables are presented as percentages and evaluated using the chi-square (χ^2^) test or the two-tailed Fischer’s exact test. Univariable analysis and multivariable analysis were used to identify risk factors for invasive GBS disease and mortality. We performed univariable analyses to compare the cases and controls in terms of risk factors. The association between independent variables is shown as the odds ratio with 95% confidence intervals, and variables for which the *P* value was less than 0.10 in the univariable analysis were included in a conditional logistic regression model for multivariable analysis. For the multivariable analysis, adjusted odds ratios (aOR) using conditional logistic regression were used to adjust for variables deemed significant by univariable analysis. The odds ratio (OR),the confidence interval (CI) and *P*-value are used to figure out if a exposure is a risk factor for EOD and LOD, and to compare the various risk factors for GBS-disease happening. An OR of greater than 1 means that there is a higher odds of disease happening with exposure to a risk factor. An OR is less than 1 is associated with lower odds. An OR of exactly 1 implies that exposure to factor does not affect the odds of disease. A two-tailed *P* value of less than 0.05 was considered to show statistical significance, and statistical analyses were performed using SPSS 19.0 (SPSS, Inc., Chicago, IL, USA).

## Results

### Incidence and risk factors of invasive neonatal GBS infection

**Fig 1.**
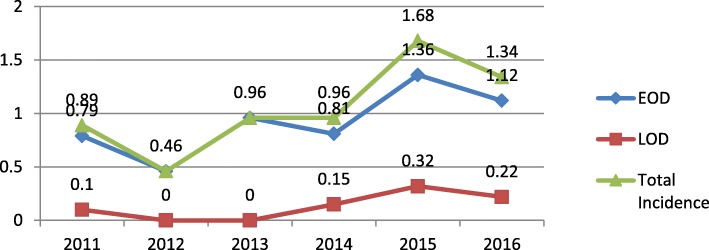
The incidence of invasive neonatal GBS infection over the 6-years

The incidence of invasive neonatal GBS infection over the 6-year study period is presented in Fig. 1.During the 6-year study period, there were 74 infants (< 90 days of age) with invasive GBS disease among 67,985 live births (LBs),yielding an overall incidence (per 1000 LBs) of invasive GBS disease of 1.09‰ (95%CI:0.81‰-1.37‰); the incidences of EOD (*n* = 65) and LOD (*n* = 9) were 0.96‰(95%CI:0.73‰-1.19‰) and 0.13‰(95%CI:0.04‰-0.22‰), respectively. There were 9 cases among 10,117 LBs in 2011(0.89/1000 LBs), 5 cases among 10,855 L Bs in2012 (0.46/1000 LBs), 10 cases among 10,463 LBs in 2013(0.96/1000 LBs), 13 cases among 13,569 LBs in 2014 (0.96/1000 LBs), 16 cases among 9552 LBs in 2015(1.68/1000 LBs), and 18 cases among 13,429 LBs in 2016(1.34/1000 LBs).

Risk factors for invasive Group B Streptococcal (GBS) disease in EOD and LOD cases and matched controls are presented in Table 1. In total 28/65 (43.1%) EOD cases occurred in either non-residents of the city or patients undergoing membrane stripping.Membrane stripping (*P* = 0.005, aOR: 3.68, 95% CI: 1.48–9.13) and non-resident mother (*P* < 0.001,aOR: 5.88, 95% CI: 2.36–14.61)were associated with a greater risk for EOD in the univariable analysis, whereas previously described risk factors such as preterm delivery (< 37 weeks’ gestational age), prolonged rupture of membranes (> = 18 h), birth weight < 2500 g, known genital GBS colonization, intrapartum fever, and non-use of IAP. According to ORs ordered by their magnitude, 5 increased risk factors found in the multivariable analysis (*P* < 0.05) ranked as follows: intrapartum temperature > =38.0 °C, GBS screened positive, non-resident mother, rupture of membranes for > = 18 h, membrane stripping.

**Table 1:** Risk factors for invasive Group B Streptococcal (GBS) disease in EOD and LOD cases and matched controls

	Cases	Controls	Univariable-OR(95%CI)	*P*-value	Multivariable-OR(95%CI)	*P*-value
EOD	(*n* = 65)	(*n* = 130)				
Non-resident mother	28	20	4.16 (2.10–8.25)	< 0.001	5.88(2.36–14.61)	< 0.001
GBS screened positive	17	5	8.85 (3.10–25.33)	< 0.001	10.97(3.02–39.88)	< 0.001
Intrapartum temperature > =38.0 °C	22	2	32.74(7.39–145.03)	< 0.001	30.78(5.37–176.53)	< 0.001
Membrane stripping	28	30	2.52 (1.33–4.78)	0.004	3.68(1.48–9.13)	0.005
Rupture of membranes for > = 18 h	22	14	4.24(1.99–9.03)	< 0.001	4.16(1.35–12.80)	0.013
No IAP	29	106	0.18 (0.09–0.35)	< 0.001	2.14(0.82–5.59)	0.122
Gestational Age < 37 weeks	17	13	3.19(1.44–7.07)	0.003	1.29(0.35–4.68)	0.701
Birth Weight < 2500 g	14	8	4.19(1.66–10.60)	0.001	2.82(0.70–11.41)	0.147
LOD	(*n* = 9)	(*n* = 27)				
Non-resident mother	4	4	4.60 (0.85–24.93)	0.086		
GBS screened positive	0	1		1.000		
Intrapartum temperature > =38.0 °C	1	0		0.250		
Membrane stripping	5	7	3.57 (0.74–17.20)	0.126		
Rupture of membranes for > = 18 h	1	1	3.25 (0.18–58.06)	0.443		
No IAP	7	26	0.14(0.01–1.71)	0.148		
Gestational Age < 37 weeks	2	2	3.57 (0.42–30.10)	0.255		
Birth Weight < 2500 g	2	3	2.29(0.32–16.51)	0.581		

All 9 cases with LOD were born to women with negative vaginal swab cultures. Although the eight identified factors were more common in cases with LOD than in controls, no increased risk was found in the chi-square analysis (*P* > 0.05).

### Clinical data on EOD and LOD cases

Comparisons between the demographic characteristics of the EOD and LOD groups are shown in Table 2. No differences were found in demographic characteristics such as gestational age, birth weight or mode of delivery between infants with EOD and those with LOD.

**Table 2:** Demographic characteristics of infants with invasive Group B Streptococcal (GBS) disease

Varibles	All cases (*n* = 74)	EOD^1^ (*n* = 65)	LOD^2^ (*n* = 9)	OR(95%CI)^3^	*p*-value^4^
Gender					
Male	33	27(41.5)	6(66.7)	0.36(0.08–1.55)	0.176
Gestational Age					
≥37 weeks	55	48(73.8)	7(77.8)	0.81(0.15–4.27)	1
< 37 weeks	19	17(26.2)	2(22.2)	1.24(0.23–6.56)	1
< 37 - ≥34 weeks	13	12(18.5)	1(11.1)	1.81 (0.21–15.89)	1
< 34 weeks	6	5(7.7)	1(11.1)	0.67(0.07–6.45)	0.554
Birth Weight					
≥2500 g	58	51(78.5)	7(77.8)	1.04 (0.19–5.58)	1
< 2500 g	16	14(21.5)	2(22.2)	0.96 (0.18–5.15)	1
1500–2499 g	15	13(20)	22(22.2)	0.88(0.16–4.72)	1
< 1500 g	1	1(1.5)	0(0)		1
Mode of delivery 1					
Vertex delivery	42	35(53.8)	7(77.8)	0.33(0.06–1.73)	0.284
Caesarean-section	28	26(40)	2(22.2)	2.33(0.45–12.13)	0.468
Obstetric forceps	4	4(6.2)	0(0)		1
Membrane sweeping	33	28(43.1)	5(55.6)	0.61(0.15–2.46)	0.501
Non-resident mother	32	28(43.1)	4(44.4)	0.95(0.23–3.85)	1

^1^ EOD-Early-onset disease

^2^ LOD-Late-onset disease

^3^ OR(95%CI)-calculated odds ratio with 95% confidence comparing EOD to LOD

^4^*p*-value-using Chi-squared, Fischer exact or Wilcoxon rank-sum (Mann-Whitney) test

Pneumonia with or without sepsis accounted 63.1% (41/65) of EOD. For infants with EOD, GBS was isolated from sputum in 34/65(52.3%), blood in 18/65(27.7%), blood and sputum in 7/65 (10.8%), blood and CSF in 1/15 (1.5%), and other samples (eye, urine, umbilicus) in 6/65(9.2%). Jaundice, asphyxia, fever, or preterm delivery were the initial symptoms for 8/65(12.3%), 5/65(7/7%), 6/65(9.2%), and 12/65(18.5%) infants with EOD, respectively. Respiratory symptoms including pneumonia at presentation (43/65, 66.2%, *P* = 0.024) or mild illness (urethritis, omphalitis, and ophthalmia) were more common among infants with EOD than among infants with LOD.

In contrast, fever at presentation (6/9, 66.7%, *P* < 0.001) was significantly more frequent among infants with LOD. Sepsis accounted 88.9% (8/9) cases of LOD. Additionally, the incidence of sepsis, shock, and meningitis was higher, though not significantly among infants with LOD. For 8/9 infants with LOD, GBS was isolated from blood, and it was isolated from sputum in 1/9.

53 /65(81.5%) infants with EOD were admitted to the NICU < 1 day after birth. 8 / 9 infants with LOD were identified at < 30 days. The median (range) infant age at presentation of EOD and LOD was 23 min (5 min-6 days) and 17 days (7-51 days), respectively (*P* < 0.001). The average length of hospitalization in NICU of EOD and LOD cases was 9.37 and 10.67 days (*P* = 0.046).

Obstetric characteristics of women whose infants developed EOD stratified by delivery method are shown in Table 3. There was no significant difference in the GBS infection rates by the three delivery modes. Among 26/74 (35.1%) women who underwent caesarean delivery and whose infant developed EOD, 25/26 (96.2%) were delivered after rupture of membranes, and the remaining woman was GBS-positive. Caesarean section was performed in response to dystocia, and 21/26 of women undergoing C-section received antibiotics. Most infants (56/74, 75.7%) were born to women with > 1 risk factor. 38/74(51.4%) women received IAP with different antibiotic dose and exposure duration before delivery. Erythromycin enteric capsule was commenced 12 h after rupture of membranes. Combined use of antibiotics can also be seen.

**Table 3:** Obstetric characteristics of women whose infants developed EOD and LOD stratified by delivery method

Mode of delivery	Membrane sweeping	Intrapartum temperature ≥ 38 °C	Rupture of membranes		Urine WBC+	Asphyxia	Intrapartum Antibiotic Prophylaxis						
			> = 18 h	< 18 h			No	erythromycin	cefazolin	cefuroxime	ampicillin	penicillin	others
EOD													
Obstetric forceps (*n* = 4)	3	1	1	1	0	3	2	0	1	0	1	0	0
Caesarean-section (*n* = 26)	10	13	15	10	5	7	5	7	5	7	0	1	1
Vaginal delivery(35)	15	8	6	12	6	9	22	5	4	1	0	3	1
LOD													
Obstetric forceps (*n* = 0)	0	0	0	0	0	0	0	0	0	0	0	0	0
Caesarean-section (*n* = 2)	1	1	1	0	0	0	1	1	0	0	0	1	0
Vaginal delivery (*n* = 7)	4	0	0	1	1	1	6	2	2	0	0	3	0

### Case fatality rate

The overall case fatality rate among cases was 8.11% (6/74), including 7.69% (5/65) for infants with EOD and 11.1% (1/9) for infants with LOD. While the difference in the mortality rate between infants with EOD and those with LOD seems large, it is not statistically significant [*P* = 0.55, OR = 0.67 (95%CI: 0.07–6.45)]. Possible predictors of mortality are shown in Table 4 but none were significant. The mothers in all 6 cases had low vaginal swabs collected at hospital admission were reported to be GBS-negative. Maternal urinary tract culture was seldom performed. Two mothers with positive urine white blood cell counts did not undergo urine culture. Only one case was GBS-positive based on CSF.GBS was detected in the blood cultures of all fatal cases with EOD. Treatment was discontinued in cases where the outcome was considered to be poor, which included a child with severe meningitis, intracranial haemorrhage, collodion baby, and prematurity with respiratory failure. The other infant died within half an hour after delivery due to asphyxia. The average length of hospitalization in NICU among fatal cases was 4.83 days, which was less than the average of 9.94 days among cured cases(*P* = 0.010). The mean birth weight among fatal and cured cases was 2.97 and 3.07 kg (*P* = 0.707), and the mean gestational age was 38.7 and 37.8 weeks (*P* = 0.42), respectively.

**Table 4:** Predictors of deaths for neonatal GBS infections

Variables	No.(%)of patients		OR(95%CI)	*P*-value
	dead (*n* = 6)	survival (*n* = 68)		
Non-resident mother	5(83.3)	36(52.9)	4.44(0.49–40.08)	0.216
Asphyxia	3(50)	17(25)	3.00(0.55–16.29)	0.334
Tracheal intubation	2(33.3)	13(19.1)	2.12(0.35–12.82)	0.595
Membrane stripping	2(33.3)	31(45.6)	0.60(0.10–3.48)	0.686
Meningitis	1(16.7)	4(5.9)	3.20(0.30–34.33)	0.353
Sepsis	5(83.3)	27(39.7)	7.59(0.84–68.61)	0.079
Intrapartum Temperature > =38.0 °C	3(50.0)	20(29.4)	2.40(0.45–12.92)	0.367
Rupture of membranes for > = 18 h	1(16.7)	21(30.9)	0.45(0.05–4.07)	0.662
No IAP	4(66.7)	30(44.1)	2.53(0.43–14.79)	0.404
GBS screened positive	0	32(47.1)		0.033
Central nervous system symptoms	3(50.0)	23(33.8)	1.96(0.36–10.47)	0.659

## Discussion

Over the past decades, GBS has been recognized as a main cause of neonatal infections associated with high morbidity and mortality [2, 5]. Striking declines in disease incidence occurred following the issuance of guidelines from the CDC for prevention of perinatal group B streptococcal disease in 1996,2002, and 2010, most likely due to GBS screening and the implementation of IAP [2, 6–8]. There are no formal prevention policies in China. It is important to identify the disease characteristics to determine the best preventive strategy. Our data reveal local incidence, risk factors, clinical characteristics and case fatality rate that could serve as a baseline report for country-specific guidelines.

Since 2001, several studies have reported invasive neonatal GBS disease in China, with one paper reporting 118 infant cases across 7 hospitals between 2011 to 2015 [9–12]. However, there is a lack of systematic research [9–12]. Our study confirms that GBS is an important pathogen in fatal, non-fatal neonatal pneumonia, sepsis with or without pneumonia especially in EOD cases [9]. Retrospective chart review plotted the incidence of neonatal GBS infection as 1.1 per 1000 live births in Taiwan, and our study found a similar incidence [10].

Zhang reported that 5 (12.5%) cases were classified as EOD and 35(87.5%) were classified as LOD [11]. In the Netherlands, only 7% of all GBS infections are LOD, which contrasts with the United Kingdom and Germany, where 33.6 and 39.2% of cases are LOD, respectively [12].In our study, the incidence of EOD and LOD was 87.8% (65/74) and 12.2% (9/74), respectively. This difference may be associated with the differences in the patient population. Fewer cases of LOD in our study may be because infants aged > 1 month may present to other local hospitals. Furthermore, receiving antibiotics at home or local community healthcare center (data is not collected) may lead to negative culture results. The higher incidence of EOD may result from limited awareness of prevention, non-standard GBS screening and IAP. When asymptomatic neonates were admitted to the NICU for prematurity, jaundice, maternal diabetes or chorioamnionitis, they received a diagnostic evaluation (blood cell count and blood/throat swab culture).This resulted in early detection, early diagnosis and early treatment.

Wortham reported that 13% of early onset infections had no documented symptoms, including 9% who remained asymptomatic at 72 h but not refer to LOD [13]. Similarly, 11 (11/67985, 0.016%) infants with GBS-positive throat swab cultures were clinically healthy and received no antibiotics, which maybe due to infants’ community and the prevalence of low pathogenic strains. Capsular polysaccharides (CPS) are thought to be associated with virulence of strains. CPS type III remains highly preponderant in cases of EOD and LOD, and CC17 possesses specific virulence traits that enhance its invasiveness [14–16]. Serotype III was the most prevalent serotype, followed by Ia, V, Ib and II. Neither serotype VII nor IX have been detected in China though it is expected that these two serotypes are associated with GBS [17]. We plan to identify the prevalent local serotype in the future.

The identification of risk factors remains a priority for prevention. Another important finding for this study is personal risk factors of EOD. In addition to previously acknowledged risk factors, we found neonatal GBS infections to be associated with membrane stripping and non-resident mother. Membrane stripping allows colonized GBS to ascend from the vagina to the amniotic fluid, and thus foetuses are exposed to GBS and aspirate the bacteria into their lungs [6, 18–21]. Especially when membrane stripping fails to result in delivery, we suggest that providers consider the use of antibiotic prophylaxis. However, when penicillin, ampicillin, cefazolin or vancomycin is administered before delivery, the dose and duration must be precise according to a specific guideline or management protocol [6]. Guidelines such as the American College of Obstetrics and Gynecologists state that the optimal timing is 4 h prior to delivery.

As a retrospective study at a single hospital, our surveillance data indicates that when a caesarean delivery is performed after membrane stripping, the risk for EOD is similar to that of a vaginal delivery. The risk for transmission is likely much higher than in caesarean delivery before rupture of membranes or onset of labour [8, 22]. Due to the vast population and high turnover, non-resident pregnant women who are low-income and without medical insurance may have poor living conditions and poor health care, which can lead to labour without prophylaxis. It is necessary to provide better management and proper medical care for non-resident women.

The overall mortality rate for neonatal GBS infections remains at approximately 10% [2]. The case fatality rate of invasive GBS disease of 8.11% is similar to published reports. In a Japanese study, the case fatality rate of EOD cases was 13.6%, which was much higher than that of LOD cases (8.0%) [23]. Recent global data show the mean rates in cases of EOD and LOD to be 12.1% (95% CI: 6.2–18.3) and 6.8%(95% CI: 4.3–9.4), respectively [2]. Prompt diagnostic evaluation will likely contribute to a lower fatality rate of EOD cases, and higher mortality of LOD cases may be due to delayed medical treatment. In contrast with Joubrel, who found lower gestational age and low birth weight to be risk factors for poor outcomes, no important predictors were found in our study [14]. Limitations of our study include GBS screened positive was not come up to the expectations. False negative results are expected because swabbing only the lower vagina decreases the culture yield substantially, and direct agar plating is used instead of selective enrichment broth [6]. This retrospective, single institution study has incomplete ascertainment of various variables that brought it limitations.The small sample size of cohort may affect the retrospective power. Fatality-related risk factors need further study due to the low prevalence of fatal cases.

## Conclusion

In conclusion, our six-year investigation identified the incidence of invasive GBS infection in the Chinese mainland. This study demonstrates remarkable country-specific variation in comparison with other countries. Many individual risk factors were discovered for acquisition of invasive group B streptococcus disease. However, our findings can improve the awareness of neonatal GBS infection and lay a cornerstone to ensure accurate representation of the burden. With China’s new two-child policy, a nationwide prevention strategy to reduce morbidity and mortality caused by GBS is urgently needed.

## Abbreviations

EOD: Early onset disease
GBS: Group B streptococcus
LOD: Late onset disease
NICU: Neonatal intensive care unit
